# Influence of Prenatal Arsenic Exposure and Newborn Sex on Global Methylation of Cord Blood DNA

**DOI:** 10.1371/journal.pone.0037147

**Published:** 2012-05-25

**Authors:** J. Richard Pilsner, Megan N. Hall, Xinhua Liu, Vesna Ilievski, Vesna Slavkovich, Diane Levy, Pam Factor-Litvak, Mahammad Yunus, Mahfuzar Rahman, Joseph H. Graziano, Mary V. Gamble

**Affiliations:** 1 Division of Environmental Health Sciences, School of Public Health and Health Sciences, University of Massachusetts, Amherst, Massachusetts, United States of America; 2 Department of Epidemiology, Mailman School of Public Health, Columbia University, New York, New York, United States of America; 3 Department of Biostatistics, Mailman School of Public Health, Columbia University, New York, New York, United States of America; 4 Department of Environmental Health Sciences, Mailman School of Public Health, Columbia University, New York, New York, United States of America; 5 International Centre for Diarrhoeal Disease Research, Bangladesh (ICDDR,B), Dhaka, Bangladesh; 6 Columbia University Arsenic Project in Bangladesh, Dhaka, Bangladesh; VU University Medical Center, The Netherlands

## Abstract

**Background:**

An emerging body of evidence indicates that early-life arsenic (As) exposure may influence the trajectory of health outcomes later in life. However, the mechanisms underlying these observations are unknown.

**Objective:**

The objective of this study was to investigate the influence of prenatal As exposure on global methylation of cord blood DNA in a study of mother/newborn pairs in Matlab, Bangladesh.

**Design:**

Maternal and cord blood DNA were available from a convenience sample of 101 mother/newborn pairs. Measures of As exposure included maternal urinary As (uAs), maternal blood As (mbAs) and cord blood As (cbAs). Several measures of global DNA methylation were assessed, including the [3H]-methyl-incorporation assay and three Pyrosequencing assays: Alu, LINE-1 and LUMA.

**Results:**

In the total sample, increasing quartiles of maternal uAs were associated with an increase in covariate-adjusted means of newborn global DNA methylation as measured by the [3H]-methyl-incorporation assay (quartile 1 (Q1) and Q2 vs. Q4; p = 0.06 and 0.04, respectively). Sex-specific linear regression analyses, while not reaching significance level of 0.05, indicated that the associations between As exposures and Alu, LINE-1 and LUMA were positive among male newborns (N = 58) but negative among female newborns (N = 43); tests for sex differences were borderline significant for the association of cbAs and mbAs with Alu (p = 0.05 and 0.09, respectively) and for the association between maternal uAs and LINE-1 (p = 0.07). Sex-specific correlations between maternal urinary creatinine and newborn methyl-incorporation, Alu and LINE-1 were also evident (p<0.05).

**Conclusions:**

These results suggest that prenatal As exposure is associated with global DNA methylation in cord blood DNA, possibly in a sex-specific manner. Arsenic-induced epigenetic modifications *in utero* may potentially influence disease outcomes later in life. Additional studies are needed to confirm these findings and to examine the persistence of DNA methylation marks over time.

## Introduction

Naturally occurring arsenic (As) is a ubiquitous element in the environment. Through processes that are incompletely understood, As in the solid phase can become solubilized and released into groundwater. Numerous aquifers around the world are enriched with this metalloid, posing a tremendous public health challenge in populations whose drinking water is obtained from contaminated aquifers [1]. Bangladesh is one of the most severely affected regions; approximately 35% of all tube wells contain concentrations of As exceeding the Bangladesh drinking water standard of 50 µg/L, and an estimated 35–77 million people are at risk for As-induced health outcomes [2].

Arsenic is classified as a human carcinogen by the International Agency for Research on Cancer and the U.S. Environmental Protection Agency [3]. However, it is often referred to as a ‘paradoxical’ human carcinogen because animal models have generally shown that As does not act as a complete carcinogen [4]. Lack of an ideal animal model due to variability in the response to As exposure across mammalian species [5] has slowed progression toward obtaining a complete mechanistic understanding of As-induced health outcomes. In 2003, Waalkes et al developed a mouse model in which brief *in utero* exposure to As induced sex-specific tumor formation in adulthood. Male offspring developed liver and adrenal tumors and female offspring developed ovarian and lung tumors [6]. Subsequent studies found aberrant gene expression profiles and alterations in hepatic DNA methylation in adult male mice with hepatocellular carcinoma induced by *in utero* As exposure [7] and in newborn mice prior to cancer development [8]. Emerging evidence in humans also suggests that early-life As exposure increases the incidence of adult-onset diseases including cancer, cardiovascular disease, and pulmonary outcomes [9], [10], [11]. A study of the effects of prenatal As exposure on gene expression profiles in newborns in Thailand reported that As exposure activated molecular pathways indicative of stress, inflammation, metal exposure and apoptosis [12]. Taken together, these data suggest that there are critical developmental windows during which As exposure increases susceptibility to adult-onset diseases, and that these effects may be mediated through persistent epigenetic changes.

The collective evidence supporting the role of As exposure in the dysregulation of DNA methylation is provided in two excellent reviews [13], [14]. For example, *in vitro* and animal studies have demonstrated that As exposure leads to reductions in global DNA methylation [8], [15], [16], [17], [18], [19], hypermethylation within p16INK4a and RASSF1A promoter regions in lung tissue [20], and hypomethylation of estrogen receptor alpha promoter in hepatic tissue [15]. In contrast to animal studies, we previously reported that, among Bangladeshi adults, As exposure was positively associated with global methylation of leukocyte DNA, and that this association was modified by plasma folate status [21]. We observed a similar relationship between As exposure and global DNA methylation among controls within a nested case-control study of As-induced skin-lesions [22] as have others in a cross-sectional study from West Bengal, India [23]. Recent *in vitro* studies have further indicated that As exposure can induce alterations in post-translational modifications of histone tails [24], [25], [26]. Together, the pleiotropic effects of epigenetic dysregulation represent a potential mechanism that may be common to multiple As-induced health outcomes.

In our study of mother/newborn pairs from 101 pregnancies in Bangladesh, we previously demonstrated strong positive correlations between maternal blood As (mbAs) and cord blood As (cbAs), indicating that As readily crosses the placenta [27]. The objective of the present analysis was to test the hypothesis that maternal As exposure influences global methylation of cord blood DNA.

## Methods

### Ethics Statement

This study was approved by the Institutional Review Board of the Columbia University Medical Center and the Ethical Review Committee of the ICDDR-B.

### Study Participants

Between December 2004 and April 2005, pregnant women who presented at the main hospital in Matlab, Bangladesh, were asked by an attending physician to participate in a study designed to characterize the As metabolic profiles among mother/newborn pairs [27]. From this parent study, the participants in the current study include 101 pregnant women, all of whom signed written informed consent. This study benefited from the International Centre for Diarrheal Disease Research-Bangladesh (icddr,b) health and demographic surveillance system (HDSS), which records all vital events, such as births, deaths, marriages, date of the last menstrual period, pregnancies and pregnancy outcomes, as well as in- and out-migration. During their pregnancies, all women were provided with daily iron (60 mg/d) and folic acid (300 µg/d) supplements.

### Sample Collection and Storage

Near the time of delivery, women were asked to provide written informed consent, to answer a brief questionnaire, to provide a blood and urine sample prior to delivery, and to allow for a blood sample to be obtained from the discarded umbilical cord after the birth of the baby. The questionnaire was administered by a trained nurse, who also obtained the blood and urine samples. The length, weight and head circumference of the baby were also recorded.

Samples were collected in EDTA vacutainers and immediately processed in the hospital laboratory. Whole blood for blood As measurements was aliquoted into acid-washed tubes. Serum was separated for folate, B_12_ and homocysteine analyses. All samples were then frozen at −80°C. Urine samples were also collected in acid-washed tubes and frozen at −80°C. All of the samples were ultimately shipped to New York on dry ice and analyzed at Columbia University.

### Water As Analyses

For 100 of the 101 women, a tube well water sample was obtained from the primary well shortly after delivery, usually within a day, into 60-mL acid cleaned polyethylene bottles. These were shipped to Columbia University's Lamont Doherty Earth Observatory where they were acidified prior to analysis. Analytical procedures have previously been described in detail [28]. All water samples were analyzed using an axiom single-collector high-resolution inductively-coupled plasma mass spectrometer (HR ICP-MS) (Thermo elemental, Erlanger, Germany). The analytical detection limit of the method is 0.1 µg As/L; the standard deviation of a single measurement is conservatively estimated at 4 µg/L for concentrations up to 150 µg/L and 2% for samples > 150 µg/L [29].

### Total Urinary and Blood As Measurements

Urinary As (uAs) concentrations were assayed by graphite furnace atomic absorption spectrophotometry (GFAA) using a Perkin-Elmer AAnalyst 600 system (Shelton, CT) as described [30]. Our laboratory participates in a quality control program coordinated by the Quebec Toxicology Center in Quebec, Canada. During the course of this study, intraclass correlation coefficients between our laboratory's values and samples calibrated at the Quebec Toxicology Center were 0.99. The detection limit of the method is 2 µg/L. In order to correct for urine dilution, we also measured urinary creatinine (uCrn) concentrations using a standard colorimetric method based on Jaffe's reaction [31] with reagents from Sigma Diagnostics (Sigma, St. Louis, MO).

Maternal and cord blood samples were analyzed for total blood As concentration using a Perkin-Elmer Elan DRC II ICP-MS equipped with an AS 93+ autosampler as described [27]. Briefly, the As concentration of the standard solution used for instrument calibration was chosen to cover the expected range of As concentrations in the blood samples: 5, 25 and 50 µg/L. Matrix suppression was compensated for by the use of iridium (Ir) as an internal standard, selected to match to the ionization potential of As. Spectral interferences for As were resolved with Dynamic Reaction Cell (DRC) technology by introducing oxygen as a second gas. The intraclass correlation coefficients between our laboratory's values and samples calibrated at the Quebec Toxicology Center were 0.99. The detection limit of the method is 0.06 µg/L.

### DNA extraction

Genomic DNA was isolated at Columbia University from 300 µl of frozen whole blood samples using the FlexiGene DNA kit (Qiagen) following the manufacturer's protocol, except for an additional centrifugation step at 10,000× g for 5 min immediately after protease digestion to pellet any remaining proteins or lipids. The supernatant was then transferred into a new microcentrifuge tube containing 150 µl of isopropanol. The remainder of the procedures were conducted according to the manufacture's protocol. All extractions yielded ≥2 µg of DNA with a 260/280 ratio ≥1.7.

### Global DNA methylation

#### Methyl Incorporation Assay

The methyl-incorporation assay was performed by the method of Balaghi and Wagner [32] as we have previously described in detail [21], [33]. DNA was incubated with [^3^H]-SAM in the presence of the SssI prokaryotic methylase enzyme, which indiscriminately methylates all unmethylated cytosines in CpG sequences. The ability of DNA to incorporate [^3^H] methyl groups *in vitro* is inversely related to endogenous DNA methylation. Briefly, 250 ng of DNA was incubated for 1 hr at 37°C with 3 U of SssI methylase (New England Biolabs, Beverly, MA), 3.8 uM (1.1 µCi) ^3^H labeled SAM (GE Healthcare), and EDTA, DTT and Tris-HCL (pH 8.2). The reaction mixtures were applied onto Whatman DE81 filter papers and washed on a vacuum filtration apparatus and air dried. Dried filters were analyzed by a Packard Tri-Carb 2100TR Liquid Scintillation Analyzer. Each DNA sample was processed in duplicate and each processing run included samples for background (reaction mixture with all components except SssI enzyme), a hypomethylation control (HeLa cell DNA) and a quality control sample (DNA extracted from a whole-blood sample). The intra- and inter-day coefficients of variation (CVs) were 1.8% and 5.3%, respectively. To quantify the amount of double-stranded input DNA in each reaction, an aliquot of the assayed DNA was used to determine DNA concentrations using PicoGreen dsDNA Quantitation Reagent (Molecular Probes). All disintegrations per minute (DPM) values were expressed per µg DNA.

#### Alu and LINE-1 methylation

DNA samples (500 ng at 20 ng/µl) were bisulfite-treated using the EZ-96 DNA Methylation-Gold Kit™ (Zymo Research, Orange, CA). Bisulfite conversion of DNA changes unmethylated cytosine to uracil and subsequently to thymidine after PCR whereas methylated cytosines are protected from bisulfite conversion, resulting in methylation-dependent differences in DNA sequences. Bisulfite-converted DNA was stored at −20°C until further use. LINE-1 and Alu methylation was measured by quantitative Pyrosequencing (Qiagen) using primers and conditions as described previously [34], [35]. Pyrosequencing employs a primer extension reaction, using a biotin-labeled single stranded PCR amplicon as template, in which pyrophosphatase (PPi) is released during the incorporation of each nucleotide in equimolar proportion to that incorporated. The level of methylation for each CpG target region was quantified using the Pyro Q-CpG Software. Human whole-genome amplified DNA and a methylated standard (Zymo Research, Orange CA) were used as 0% and 100% methylated controls, respectively. Inter-day CVs for Alu and LINE-1 were 2.0% and 0.8%, respectively.

#### LUMA

The Luminometric Methylation Assay (LUMA) has been described in detail elsewhere [36], [37]. LUMA is based on recognition and cleavage of 5′- CCGG -3′ sequences by the methylation sensitive restriction enzyme (HpaII) and its methylation insensitive isoschizomer (MspI) in parallel reactions. Additionally, EcoRI is included in all reactions to normalize the amount of DNA input. The extent of restriction cleavage is then measured by bioluminometic polymerase extension via Pyrosequencing.

Briefly, genomic DNA (300 ng) was digested with HpaII+EcoRI and MspI+EcoRI in parallel reactions containing 2 µl of Tango buffer (Fermentas, Glen Burnie, MD), 5 U of HpaII or MspI, 5 U of EcoRI and dH2O to a final volume of 20 µl. Samples were incubated at 37°C for 4 hours. After the incubation, 15 µl of Pyrosequencing annealing buffer was added to samples. After mixing, 30 µl of the sample reaction containing the annealing buffer was transferred to 96 well Pyrosequencing plates and run with the following nucleotide dispensation order: GTGTCACATGTGTG [36], [38]. Peak heights of nucleotide incorporation from the resulting pyrograms were used to calculate % global DNA methylation using the formula: 1−[(HpaII(G)/EcoRI(T))/(MspI(G)/EcoRI(T))]×100, where G and T are the peak heights for HpaII or MspI (methylation) and EcoRI (input DNA), respectively. All samples were run in duplicate. The intra- and inter-day CVs were 0.55% and 3.01%, respectively.

### Plasma Folate, Cobalamin and Homocysteine

Plasma folate and total cobalamin from maternal and cord blood were analyzed by radioimmunoassay (Quantaphase II, Bio-Rad Laboratories, Richmond CA) as described previously [39]. The intra- and inter-day CVs for folate were 3% and 11%, respectively, and those for cobalamin were 4% and 8%, respectively. Plasma tHcys concentrations were measured by HPLC with fluorescence detection according to the method described by Pfeiffer et al [40]. The intra- and inter-day CVs for tHcys were 5% and 8%, respectively.

### Statistical Analyses

Descriptive statistics were calculated for sample characteristics of mothers and their newborns and by newborns' sex. Sex differences for continuous variables were assessed using the Wilcoxon rank-sum test. Pyrosequencing data that did not pass Pyro Q-CpG Software quality scores or were not reproducible were excluded from analyses; these included three samples (one maternal Alu, one newborn Alu and one newborn LINE-1). For the methyl-incorporation assay, quality data was available for 98 samples. Spearman correlation coefficients were used to assess bivariate associations between maternal and newborn DNA methylation and sex specific correlations between uCrn and DNA methylation measures. Wald tests were used to evaluate differences in correlations by sex. Linear regression models were used to examine associations between As exposures and DNA methylation measures controlling for uCrn, including a uCrn by sex interaction term as appropriate. An As by sex interaction term was added to test for sex differences in the effect of As on DNA methylation. We also categorized uAs adjusted for uCrn, defined as the residual from regressing log uAs on sex-specific log uCrn, into quartiles, and computed mean levels of global DNA methylation for each quartile. All analyses were performed using SAS (version 9.2; SAS Institute Inc., Cary, NC).

## Results

The demographic and clinical data of the study population are presented in **Table 1**. The mean age of the women was 26.7±5.3 years and mean parity was 1.8±0.9. Of the 101 births, 58 were males. The average birth weight was higher among male compared to female newborns (2,830.5±517.2 g vs 2,647.0±336.5 g; p<0.05). Gestational age did not differ between male and female newborns. Water As concentrations ranged from 0.01 to 661 µg/L; 28% and 38% of tube wells exceeded the Bangladesh standard of 50 µg/L and the World Health Organization guideline of 10 µg/L, respectively. As previously reported [27], there was a strong correlation between cbAs and mbAs concentrations (r = 0.84; p<0.0001), with the mean cbAs concentration higher than the mean mbAs concentration (15.7 µg/L vs. 11.9 µg/L, respectively; p<0.0001). There were no significant differences in As exposures by newborn sex. As compared to male newborns, female newborns had higher maternal plasma homocysteine (6.7±1.5 µmol/L vs. 6.1±1.6 µmol/L; p = 0.04) and cord plasma homocysteine (6.1±1.4 µmol/L vs. 5.4±1.3 µmol/L; p = 0.008). None of the As exposure variables were significantly associated with gestational age, birth weight and length, or head circumference; however, male newborns had higher placenta and birth weight and larger head circumference as compared to female newborns (p<0.05).

**Table 1:** Demographics and clinical data of the study population.

	Mother^a^	Newborn^a^	
Variable	(N = 101)	Male (N = 58)	Female (N = 43)
**Maternal**			
Age (years)	26.7 (5.3)	27.5 (5.4)	25.7 (5.0)
Education (years)	7.4 (3.6)	7.6 (3.5)	7.2 (3.7)
Parity	1.8 (0.90)	1.9 (0.8)	1.7 (0.9)
Water arsenic (µg/L)	90.5 (165.8)^b^	92.5 (157.0)	87.8 (179.2)^h^
Urinary arsenic (µg/L)	127.6 (232.9)	134.3 (226.1)	118.7 (244.3)
Urinary creatinine (mg/dl)	48.6 (35.3)	47.6 (32.9)	50.1 (38.7)
Urinary arsenic (ug/g creatinine)	271.7 (489.5)	282.1 (450.3)	257.7 (543.1)
Blood arsenic (µg/L)	11.9 (8.6)	12.6 (10.1)	10.9 (6.0)
Plasma folate (nmol/L)	20.3 (9.9)^c^	20.7 (79.6)	19.6 (9.4)^i^
Plasma homocysteine (µmol/L)	6.4 (1.5)	6.1 (1.6)	6.7 (1.5)*
Plasma B_12_ (pmol/L)	180.8 (71.5)^d^	193.6 (79.6)	163.2 (54.9)^j^
**Newborn**			
Gestational age (days)		275.9 (18.1)	281.7 (13.9)
Placenta weight (g)		531.4 (127.9)	489.5 (106.4)*
Birth weight (g)		2830.5 (517.2)	2647.0 (336.5)*
Head circumference (cm)		32.1 (3.0)^e^	31.2 (1.4)*
Birth length (cm)		48.2 (3.5)	47.7 (1.9)^j^
Cord plasma folate		52.4 (17.8)^g^	53.2 (15.3)^h^
Cord plasma homocysteine (µmol/L)		5.4 (1.3)^e^	6.1 (1.4)**
Cord plasma B12 (pmol/L)		409.4 (255)^g^	313.4 (150.0)^k^
Cord blood As (µg/L)		16.0 (9.9)	15.3 (6.9)
**DNA methylation**			
Methyl-incorporation (DPM/ug DNA)	91,718 (15,300)	89,024 (12,171)^f^	89,915 (13,861)^h^
Alu (%)	24.70 (0.81)^b^	24.76 (1.2)^e^	24.69 (1.0)
LINE-1 (%)	79.12 (1.38)	79.58 (1.8)	78.82 (1.4)^h^ **
LUMA (%)	74.72 (2.48)	74.88 (2.6)	74.86 (3.7)

a Values are mean (SD).

b N = 100,

c N = 99,

d N = 95,

e N = 57,

f N = 56,

g N = 54,

h N = 42,

i N = 41,

j N = 40,

k N = 37.

* P<0.05; Wilcoxon rank sum test for differences between newborn sex.

** P<0.01; Wilcoxon rank sum test for differences between newborn sex.

Global DNA methylation levels for the methyl-incorporation, Alu, LINE-1 and LUMA assays for mothers and newborns are also provided in Table 1 (full methylation data are available on request for academic or non-commercial purposes). Data are expressed as the percent of DNA methylation for Alu, Line-1 and LUMA. For the methyl-incorporation assay, the data are expressed as disintegrations per minute per microgram DNA (DPM/µg DNA), and are inversely related to endogenous DNA methylation levels. LINE-1 methylation was higher in male compared to female newborns (79.58±1.8 vs. 78.82±1.4; p = 0.001). The associations between mother/newborn pairs for each DNA methylation assay are shown in **Table 2**. Positive associations between mother/newborn pairs were observed for DNA methylation as measured by methyl-incorporation (Spearman correlation coefficient: r = 0.32, p = 0.001), LUMA (r = 0.22, p = 0.03), and LINE-1 (r = 0.21, p = 0.03). The correlation between mother/newborn pairs for Alu methylation was not statistically significant. In newborn sex-specific analyses, the associations between mother/newborn pairs for the methyl-incorporation assay were similar among male (r = 0.35, p = 0.007) and female (r = 0.31, p = 0.047) newborns. For the three pyrosequencing assays; however, the associations between maternal/newborn DNA methylation levels differed by newborn sex. For example, the correlations between mother/newborn pairs were significant for Alu methylation and LUMA among female newborns (r = 0.42, p = 0.005 and r = 0.37, p = 0.02, respectively), but not among male newborns. In contrast, a significant correlation for LINE-1 methylation was apparent only among male newborns (r = 0.39, p = 0.002).

**Table 2:** Spearman correlation coefficients for global DNA methylation between maternal vs. newborn pairs, combined and stratified by newborn sex.

	Methyl-incorporation	Alu	LINE-1	LUMA
	r (n)	r (n)	r (n)	r (n)
Total Sample	0.32 (98)**	0.15 (99)	0.21 (100)*	0.22 (101)*
Male newborns	0.35 (56)**	−0.05 (57)	0.39 (58)**	0.12 (58)
Female newborns	0.31 (42)*	0.42 (42)**	−0.02 (42)	0.37 (43)*

* p<0.05;

** p<0.01.

**Figure 1:**
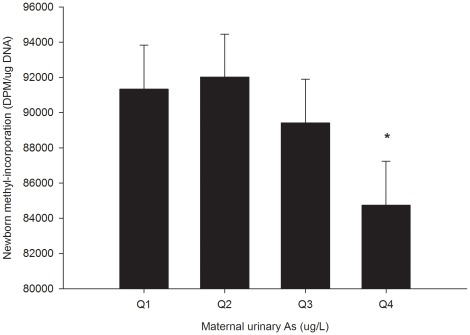
Newborn global DNA methylation and maternal urinary As exposure. Mean (SE) newborn global methylation levels as measured by the methyl-incorporation assay by quartiles of maternal uAs exposure adjusted for the sex-specific effect of uCrn. * Q1 and Q2 vs. Q4; p = 0.06 and 0.04, respectively.

No significant correlations were observed between As exposure and maternal and newborn DNA methylation (**Table S1**). However, in linear regression models adjusting for sex-specific effects of maternal uCrn levels, maternal uAs was negatively related with newborn methyl-incorporation levels (**Table 3**: b = −2049±1087; p = 0.06). **Figure 1** shows the pattern of the mean level of newborn DNA methylation, measured by the methyl-incorporation assay, among quartiles of maternal uAs adjusted for the sex-specific effect of uCrn. The mean newborn methyl-incorporation levels tended to decrease with increasing maternal uAs, indicating that uAs is associated with an increase in global DNA methylation. Compared to the first and second lowest quartiles of maternal uAs, newborns in the highest quartile of maternal uAs had lower methyl incorporation levels (Q1: 91,331±2,499 DPM/µg DNA and Q2: 92,021±2,436 DPM/µg DNA vs. Q4: 84,745±2,491 DPM/µg DNA (p = 0.06 and 0.04, respectively). Maternal uAs was not significantly associated with newborn methylation of Alu, LINE-1 and LUMA in the total sample (**Table 3**). We also found no significant associations between mbAs or cbAs concentrations and measures of newborn DNA methylation in the total sample.

**Table 3:** Estimated parameter for associations between biomarkers of As exposure and newborn global DNA methylation assays combined and stratified by sex.

	DPM/µg DNA	Alu	LINE-1	LUMA
	b ± se (p-value)	b ± se (p-value)	b ± se (p-value)	b ± se (p-value)
**Total Sample** 1				
Water As	−196.1±412 (0.64)	−0.0004±0.04 (0.99)	−0.016±0.05 (0.76)	−0.126±0.10 (0.21)
mbAs	−1858.2±2478 (0.46)	−0.035±0.22 (0.87)	0.189±0.32 (0.55)	0.267±0.60 (0.66)
cbAs	−1627.3±2417 (0.50)	−0.046±0.21 (0.83)	0.201±0.32 (0.53)	0.060±0.60 (0.92)
uAs	−2049.2±1087 (0.06)	−0.003±0.10 (0.98)	−0.022±0.14 (0.87)	0.009±0.27 (0.71)
**Female cord blood**				
Water As	334.9±668 (0.62)	−0.0378±0.05 (0.45)	−0.050±0.07 (0.50)	−0.311±0.19 (0.12)
mbAs	−2902.5±3834 (0.45)	−0.464±0.29 (0.12)	−0.266±0.43 (0.54)	−0.530±1.12 (0.64)
cbAs	−2037.1±3507 (0.56)	−0.458±0.27 (0.09)	−0.053±0.41 (0.90)	−0.445±1.02 (0.66)
uAs	−1771.1±1657 (0.29)	−0.164±0.13 (0.21)	−0.314±0.18 (0.09)	−0.218±0.49 (0.66)
**Male cord blood**				
Water As	−416.0±526 (0.43)	0.035±0.05 (0.48)	0.047±0.07 (0.53)	0.005±0.12 (0.97)
mbAs	−1063.5±3274 (0.75)	0.283±0.30 (0.35)	0.515±0.45 (0.25)	0.827±0.68 (0.23)
cbAs	−1227.7+3379 (0.72)	0.355±0.31 (0.26)	0.430±0.47 (0.36)	0.553±0.71 (0.44)
uAs	−2279.0±1454 (0.13)	0.123±0.14 (0.37)	0.196±0.20 (0.33)	0.330±0.30 (0.28)
**Total sample** 1 **^,^** 2				
Water As x sex	−871.3±842 (0.30)	0.039±0.07 (0.60)	0.085±0.11 (0.44)	0.289±0.20 (0.16)
mbAs x sex	1839.0±5026 (0.72)	0.747±0.43 (0.09)	0.781±0.64 (0.23)	1.426±1.22 (0.25)
cbAs x sex	809.5±4860 (0.87)	0.813±0.42 (0.05)	0.482±0.63 (0.45)	1.032±1.20 (0.39)
uAs x sex	−507.9±2195 (0.82)	0.288±0.19 (0.14)	0.510±0.28 (0.07)	0.553±0.47 (0.24)

1 Sex- specific effect of uCrn was controlled in the models for DPM/DNA, Alu and Line-1.

2 Interaction by newborn sex.

In secondary analyses, we observed sex-specific patterns of association between As exposure and newborn DNA methylation via pyrosequencing (**Table 3**). Arsenic exposure measurements, while not statistically significant, were negatively related to the DNA methylation measures, Alu, LINE-1, and LUMA, for female newborns (N = 43), whereas these relationships were positive for male newborns (N = 58). Moreover, we observed borderline significant sex differences in the associations between blood As levels, cbAs and mbAs, and Alu methylation (p = 0.05 and 0.09, respectively) and between maternal uAs and LINE-1 methylation (p = 0.07). The methyl-incorporation assay did not display a sex-specific pattern of association with As exposure.

While not part of our a priori hypothesis, we observed an unexpected relationship between maternal uCrn levels and newborn global DNA methylation dependent on newborn sex (**Table 4**). Among male newborns, maternal uCrn was positively correlated with global DNA methylation (methyl-incorporation: r = −0.12, p = 0.38; Alu: r = 0.34, p<0.01 and LINE-1: r = 0.28, p<0.05). In contrast, among female newborns, maternal uCrn was negatively related with DNA methylation (methyl-incorporation: r = 0.49, p<0.01; Alu: r = −0.06, p = 0.69; and LINE-1: r = −0.14, p = 0.36). We also found that these sex-specific patterns of association between maternal uCrn and newborn DNA methylation measures, i.e.methyl-incorporation, Alu and LINE-1 methylation, were statistically different (p<0.05). The sex difference in the associations of uCrn with methyl-incorporation and LINE-1 methylation were still evident in linear regression models when uAs was included in the model (p = 0.002 and  = 0.07, respectively). A similar sex-specific pattern of association was evident between cord blood homocysteine and newborn DNA methylation measures, although only LINE-1 methylation reached statistical significance (p<0.05). Sex-specific patterns were not evident between other indicators of one-carbon metabolism and newborn DNA methylation.

**Table 4:** Spearman correlation coefficients for newborn global DNA methylation vs. urinary creatinine and indicators of one-carbon metabolism in plasma, combined and stratified by newborn sex.

	Methyl-incorporation	Alu	LINE-1	LUMA
	r (n)	r (n)	r (n)	r (n)
**Total Sample**				
Maternal urinary creatinine	−0.16 (98)	0.16 (100)	0.14 (100)	−0.16 (101)
Maternal folate	0.06 (96)	−0.04 (98)	−0.11 (98)	0.08 (99)
Maternal B12	−0.05 (92)	−0.09 (94)	−0.03 (94)	0.05 (95)
Maternal homocysteine	0.06 (98)	0.06 (100)	−0.01 (100)	0.07 (101)
Cord folate	0.03 (93)	−0.18 (95)	−0.16 (95)	0.02 (96)
Cord B12	0.02 (89)	−0.09 (90)	−0.23 (90)*	−0.003 (91)
Cord homocysteine	0 (97)	0.03 (99)	−0.07 (99)	−0.05 (100)
**Male newborns**				
Maternal urinary creatinine	−0.12 (56)	0.34 (57)**	0.28 (58)*	−0.12 (58)
Maternal folate	0.23 (56)	−0.10 (57)	−0.19 (58)	0.11 (58)
Maternal B12	−0.02 (53)	−0.20 (54)	−0.19 (55)	−0.11 (55)
Maternal homocysteine	−0.04 (56)	0.10 (57)	0.08 (58)	0.14 (58)
Cord folate	0.09 (52)	−0.31 (53)*	−0.25 (54)	0.03 (54)
Cord B12	0.08 (53)	−0.21 (53)	−0.32 (54)*	−0.10 (54)
Cord homocysteine	−0.08 (55)	0.12 (56)	0.14 (57)	0.03 (57)
**Female newborns**				
Maternal urinary creatinine	0.49 (42)** ^,^ b	−0.06 (43)^a^	−0.14 (42)^a^	−0.15 (43)
Maternal folate	−0.17 (40)	0.02 (41)	0.01 (40)	0.10 (41)
Maternal B12	−0.04 (39)	0.05 (40)	0.12 (39)	0.32 (40)*
Maternal homocysteine	0.14 (42)	0.04 (42)	−0.04 (42)	−0.07 (43)
Cord folate	−0.08 (41)	−0.01 (42)	0.01 (41)	0.01 (42)
Cord B12	−0.03 (36)	0.10 (37)	−0.27 (36)	0.20 (37)
Cord homocysteine	0.07 (42)	−0.12 (43)	−0.27 (42)^a^	−0.28 (43)

* p<0.05;

** p<0.01.

a p<0.05;

b p<0.01 from Wald test for sex differences in correlations.

## Discussion

The objective of this study was to investigate the associations between prenatal As exposure and global methylation of cord blood DNA. In the total sample, our data indicated that maternal uAs was positively related to global methylation of cord blood DNA as assessed by the methyl-incorporation assay. In secondary analyses, we observed sex-specific patterns of association between As exposure and DNA methylation. Among male newborns, there was a consistent positive association between As exposure and DNA methylation. In contrast, As exposure was generally inversely associated with DNA methylation among female newborns. To our knowledge, there are no previous mother/newborn studies in humans investigating the association between prenatal As exposure and global methylation of cord blood DNA.

Growing evidence in human and animal studies suggests that early-life exposure to As increases the incidence of adverse health outcomes later in life, despite a reduction or elimination of exposure during adulthood. For example, inhabitants of Chile's region II who were born just prior to or during an acute increase in water As concentrations, resulting in early-life and prenatal exposure, had higher mortality rates from malignant and nonmalignant lung disease and acute myocardial infarction as young adults compared to unexposed individuals from a different region [9], [10], [11]. The data also indicated that sex modified the effects of early-life As exposure and subsequent mortality rates, such that males had higher mortality rates from lung cancer and acute myocardial infarction than females. Likewise, animal models have shown that brief prenatal exposure produced tumor formation in adulthood in a sex-specific manner [6]. Interestingly, tumor-free newborn mice, subjected to the same prenatal exposure protocol, exhibited similar changes in hepatic gene expression as observed in adult mice with hepatic tumors as well as reduced hepatic DNA methylation within GC-rich regions [8]. These studies indicates that epigenetic processes mediate persistent changes in gene expression and that newborn sex modifies the incidence of adult-onset diseases in response to early-life As exposure. Our data is consistent with the possibility that sex-specific epigenetic effects of As exposure could induce differential health outcomes between males and females later in life.

Overall, our results indicate that maternal uAs is positively associated with newborn global DNA methylation as assessed by the methyl-incorporation assay. Previous studies from our group have demonstrated that uAs concentrations were associated with an increase in global DNA methylation among Bangladeshi adults [21], [22]. Similarly, As-associated increases in global DNA methylation were found among adults from West Bengal, India [23]. While animal and cell culture experiments have shown that As exposure is associated with global DNA hypomethylation [8], [15], [16],[17],[18],[19], our contradictory findings may be a result of differences in tissues/cells analyzed and duration and/or magnitude of exposure [21].

Our previous studies also found that plasma folate modified the association between As exposure and global DNA methylation, such that only participates with adequate folate status (≥9 nmol/L) showed a positive association [21], [22]. In the current study, most of the women in the study were taking prenatal vitamin supplements; therefore, plasma concentrations of folate and B12 are reflective of recent supplement use rather than long-term nutritional status. While B12 was found to be inversely correlated with LINE-1 methylation, we consider the direction of the correlation to be opposite to what one might have predicted and may have been a chance observation. Adjusting for folate and other covariates did not influence the magnitude of this effect (data not shown).

In secondary analyses, we found that newborn sex modified the association between As exposure and global methylation of cord blood DNA. Although the associations did not reach statistical significance, arsenic exposure and DNA methylation were positively associated in males and inversely associated in females. These results were unanticipated and were not part of our a priori hypothesis. However, a growing body of evidence in humans suggests that sex differences may arise in epigenetic responses to prenatal environmental exposures [41]. For example, the Dutch Hunger Winter Families Study found prenatal exposure to famine was associated with sex-specific DNA methylation changes that persisted into adulthood [42]. It has been proposed that sex-specific epigenetic differences may be due to sex hormones and their effects on organ development [41]. Although limited data exists on hormonal regulation of epigenetic processes during the neonatal period, progesterone and estrogen have been shown to downregulate DNA methyltransferase expression in women [43], and higher methylation of repetitive sequences has been reported in adult men as compared to women [44], [45]. We also observed that male newborns had significantly higher LINE-1 methylation in cord blood as compared to female newborns. Interestingly, a recent study in adult mice reported sex-specific changes in hepatic global DNA methylation. In response to As exposure and a methyl-deficient diet, female mice had a significant increase in DNA methylation whereas male mice had a decrease in DNA methylation [46].

Our findings for sex-specific associations between maternal uCrn and global DNA methylation in cord blood were unanticipated. Numerous studies from our group have showed that uCrn is a strong predictor of As methylation [27], [47], [48], [49]. Moreover, uCrn was found to be an independent predictor of the risk for development of As-induced skin lesions, such that every doubling of uCrn concentrations was associated with 60% reduced risk for skin lesion development [22]. The final step in creatine biosynthesis is a methylation reaction which consumes roughly 50% of all SAM [50], [51], and endogenous creatine synthesis is downregulated by dietary creatine intake. Creatine, together with several isoforms of creatine kinase, plays critical roles in many aspects of energy metabolism in tissues with high energy requirements [52]. Because creatine is nonenzymatically degraded to creatinine, which is excreted in urine at a constant rate, uCrn is commonly used to normalize for fluctuations in the concentration of urine. However, uCrn concentrations are strongly influenced by other factors such as dietary creatine intake from protein sources, age, sex, and muscle mass [53], [54]. Although little is known about fetal requirements for creatine, animal studies indicate that the creatine transporter, responsible for cellular creatine uptake, is widely expressed very early in development, whereas fetal creatine biosynthesis doesn't occur until very late in pregnancy, suggesting that the developing fetus is dependent on transport of maternal creatine across the placenta [55]. It is unclear why we observed sex-specific patterns of association (e.g., positive in male and negative in female newborns) between DNA methylation and maternal uCrn levels. Creatine biosynthesis is differentially influenced by estrogen and testosterone [52], and maternal circulating sex hormone concentrations are influenced by the sex of the fetus [56]. Thus, although speculative, it is possible that maternal creatine biosynthesis is influenced by fetal hormone levels. Because estrogen levels may be associated with both creatinine and DNA methylation, it is possible that the sex-specific association between maternal uCrn and cbDNA methylation could be explained by estrogen. However, we cannot rule out the possibility that this was simply a chance observation, and additional studies are needed to replicate this unexpected finding.

An advantage of this study is the use of multiple assays to estimate global DNA methylation levels. Although the four methods have been used widely as indicators of global DNA methylation [32], [38], [57], the genomic targets for each assay are distinct. They can be classified as either genome-wide assays (methyl-incorporation and LUMA) or as repetitive element assays (Alu and LINE-1). Of the four assays, the methyl-incorporation assay provides the greatest genomic coverage, as it measures all unmethylated CpG sites across the genome. Of note, this reaction (using unlabeled S-adenosylmethionine) is routinely used to generate 100% methylated DNA standards for other DNA methylation techniques, such as Pyrosequencing [58]. Using the methyl-incorporation assay, DNA methylation was positively associated with maternal uAs levels in our total study sample and also showed the strongest correlation between mother and newborn DNA methylation levels. LUMA is another genome-wide assay that employs the methyl-sensitive and insensitive enzymes, HpaII and MspI, respectively, to interrogate the methylation status of the estimated 2.2 million CpG dinucleotides contained within CCGG sequences in the human genome [59]. LUMA has been previously shown to be associated with environmental exposures [36]. However, only roughly 8% of total genomic CpGs are located within CCGG HpaII recognition sequence [59], thus the leaner coverage may explain why our results for LUMA were not as robust as our findings for the methyl incorporation assay.

Alu and LINE-1 are highly repetitive elements. Approximately 1.5 million copies of Alu and 0.5 million copies of LINE-1 are dispersed throughout the genome. Together they comprise around 25% of the genome and > 40% of methylated CpG domains [34], [60]. As such, Alu and LINE-1 methylation are considered to be surrogates for global DNA methylation and have been found to be associated with a number of environmental exposures [35], [61], [62]. These methylation analyses are based on PCR amplification of consensus LINE-1 and Alu sequences, which amplifies a representative subset of elements (e.g. approximately 1% of Alu elements [57]). Downstream sequencing is then limited to three (Alu) and four (LINE-1) consecutive CpG dinucleotides. In our study, Alu and LINE-1 methylation display a distinct sex difference in association with As exposure. It is unclear if these differential associations are influenced by sex hormones of the developing fetus or other unidentified factors.

An unanticipated finding was that the associations between maternal DNA methylation and that of her newborn as measured by Pyrosequencing differed by newborn sex, such that LINE-1 methylation was correlated only among male newborn/maternal pairs, whereas Alu methylation was correlated among female newborn/maternal pairs. These inconsistencies complicate the interpretation of Alu and LINE-1 as surrogates for global DNA methylation. A potential explanation for these differences may be related to the fact that Alu and LINE-1 use different types of internal RNA polymerase promoters, suggesting that these repetitive elements may be under different selective pressures [63]. Differences in copy numbers within sex chromosomes may also contribute to these inconsistencies. It has also been reported that higher annealing temperatures produced larger differences in LINE-1 methylation levels between sexes [45].

A limitation to this study is our use of a convenience sample of participants drawn from a study that was originally designed to characterize the As metabolite profiles among only 101 mother/newborn pairs [27]. This resulted in low statistical power for the current study, especially after stratifying our data by sex or testing for interactions. Although this impaired our ability to detect statistically significant associations, the data are strongly suggestive of an influence of maternal As exposure on cord blood DNA methylation that may differ by sex.

In conclusion, the results of this study suggest that newborn sex may modify the association between prenatal As exposure and global methylation of cord blood DNA. Our data offer a plausible epigenetic mechanism by which prenatal As exposure may contribute to observed sex differences in As-induced health outcomes later in life. Additional studies are needed to confirm these results and to examine the persistence of DNA methylation marks across the lifecourse.

## Supporting Information

Table S1 **Spearman correlation coefficients between As exposures and maternal and newborn DNA methylation.**
(DOCX)
