# Supplementary material for: Influence of Prenatal Arsenic Exposure and Newborn Sex on Global Methylation of Cord Blood DNA
Source: PLoS One. 2012 May 25;7(5):e37147. doi: 10.1371/journal.pone.0037147 (PMC3360698; doi:10.1371/journal.pone.0037147)
Supplement: Table S1 — Spearman correlation coefficients between As exposures and maternal and newborn DNA methylation. (DOCX) [file pone.0037147.s001.docx]

|  | **Mother** | | | | **Newborn** | | | |
| --- | --- | --- | --- | --- | --- | --- | --- | --- |
| **Variable** | **Methyl-incorporation** | **Alu** | **LINE-1** | **LUMA** | **Methyl-incorporation** | **Alu** | **LINE-1** | **LUMA** |
| Water As | 0.149 | -0.158 | 0.018 | -0.035 | 0.048 | 0.058 | 0.006 | -0.057 |
| Maternal urinary As/Cr | 0.027 | -0.054 | -0.004 | -0.048 | -0.173 | 0.0527 | 0.020 | 0.014 |
| Maternal blood As | 0.035 | -0.117 | 0.095 | 0.113 | 0.009 | 0.096 | 0.127 | 0.070 |
| Cord blood As |  |  |  |  | -0.027 | 0.068 | 0.103 | 0.040 |
